# Getting to grips with wildlife research by citizen scientists: What role for regulation?

**DOI:** 10.1002/pan3.10151

**Published:** 2020-10-06

**Authors:** Alexandra Palmer, S. James Reynolds, Julie Lane, Roger Dickey, Beth Greenhough

**Affiliations:** 1 School of Geography and the Environment, University of Oxford, Oxford, UK; 2 School of Biosciences, University of Birmingham, Birmingham, UK; 3 Animal Welfare Ethical Review Board (AWERB), University of Birmingham, Birmingham, UK; 4 Army Ornithological Society (AOS), Aldershot, UK; 5 National Wildlife Management Centre, Animal and Plant Health Agency, York, UK

**Keywords:** animal welfare, biotelemetry, capture, citizen science, conservation, ethics, handling, trapping

## Abstract

1. Wildlife research by citizen scientists, involving the capture and handling of animals, provides clear scientific benefits, but also potential risks to animal welfare. We explore debates about how best to regulate such work to ensure that it is undertaken in an ethical manner.
2. We focus on the UK as a case study, drawing on qualitative research and stakeholder engagement events. We show that because trapping and marking of certain species requires minimal licensing, training and justification, some argue for increased formal regulation to minimise risks to animal welfare. However, others have reflected on the already complex regulatory landscape affecting wildlife research, and have expressed concern that introducing additional formal regulations could potentially make citizen science working with wildlife more difficult. Informal regulation could therefore offer a preferable alternative.
3. We set out three steps that could be taken to open up conversations about ethics and regulation of wildlife-focussed citizen science, in the UK and elsewhere: (a) take stock of wildlife-focussed citizen science in terms of numbers and harms to animal welfare; (b) assess the state of formal regulations and consider reforms; and (c) consider informal regulations as alternatives or additions to formal regulations.

## 1 Introduction

There is a long history, in the UK and elsewhere, of public participation in data collection for science, including wildlife recording. Nowadays, such work is commonly referred to as ‘citizen science’ (Cooper & Lewenstein, 2016; Eitzel et al., 2017). Although this is a term that had been used previously, it was formally conceptualised by the sociologist Alan Irwin in 1995 in the UK and by the ornithologist Rick Bonney in 1996 in the USA. As Cooper and Lewenstein (2016) summarise, Irwin’s definition described a kind of ‘democratic citizen science’, emphasising the need to open up science processes and policy to the public. In contrast, Bonney’s described ‘participatory citizen science’, in which members of the public contribute observations or efforts for scientific research (see also Eitzel et al., 2017; Riesch & Potter, 2014). Bonney’s concept has become the more widely used of the two, although recently the term has increasingly incorporated elements of both ideas (Cooper & Lewenstein, 2016). Approaches that lean towards a democratic approach might closely resemble participatory action research (PAR), which involves collaboration between academic researchers and participants to examine and improve a problematic situation for the participants (Eitzel et al., 2017; Kindon et al., 2009). Developing this still further, what has been termed ‘extreme citizen science’ might involve scientists acting as facilitators who help participants to achieve their goals; or, the entire process could be carried out without professional scientists (Haklay, 2013). Citizen science projects therefore potentially have multiple goals, such as scientific data collection, fostering public engagement with nature or science, and social change. Use of the term ‘citizen science’ may also vary cross-culturally, in part because the term may not be the most appropriate to describe indigenous peoples’ knowledge and involvement in scientific data collection (Eitzel et al., 2017). Indigenous knowledge holders might be important ‘citizen scientists’, such as in participatory mapping (Kimura & Kinchy, 2016) and wildlife and environmental monitoring projects (Flora & Andersen, 2016; Hoyte, 2017) aimed at helping indigenous groups access territorial and resource rights. Indigenous knowledge may also be sought for environmental research and management, such as in the field of ethnobiology (Anderson et al., 2012). However, as Eitzel et al. (2017) observed, the term ‘science’ may not always be appropriate or acceptable to these groups, although indigenous knowledge development might also be regarded as an example of science in its own right (see for example Giraldo Herrera, 2018). Eitzel et al. (2017) also observed that referring to indigenous peoples as ‘citizens’ may in some cases be inadvisable due to the legacies of colonialism.

For our purposes, we use the term ‘citizen science’ with reference to the participation by members of the public in wildlife research, irrespective of the more specific goals of the project or the citizen scientists’ level of involvement in determining the research methods, goals, and outcomes. By ‘members of the public’ we mean people who are not employed, working towards qualifications, or in possession of formal qualifications in wildlife research. We recognise that these terms and working definitions may not be appropriate for discussing the involvement of indigenous peoples in scientific knowledge production. However, our paper primarily focuses on the UK, where the term ‘citizen science’ is widely used (Eitzel et al., 2017) and which lacks a recent history of colonisation.

The field of astronomy perhaps boasts the highest profile citizen science projects, many of which have used the platform Zooniverse to collect volunteer observations of celestial objects and phenomena (e.g. Faherty et al., 2020). Alongside astronomy, wildlife research tends to dominate in the public profile of citizen science, perhaps because such work lends itself so readily to public participation (Dickinson et al., 2010; Roy et al., 2012). While citizen science focussed on wildlife takes many forms, including surveys that involve minimal disturbance, we write specifically about activities involving handling or other direct interaction—for example, trapping, marking and the use of tracking devices—with free-ranging wild animals. Hereafter, we refer to such work simply as ‘wildlife-focussed citizen science’. Examples of such projects in the UK include the National Nathusius’ Pipistrelle Project (Bat Conservation Trust, 2020) and ringing schemes of the British Trust for Ornithology (BTO; Robinson et al., 2019), which both enlist experienced ringers to provide species survey data. Another example is the Mammal Society’s research comparing methods for monitoring harvest mice (*Micromys minutus*) and other small mammals, which involved live trapping undertaken by non-professional volunteers (Poulton & Turner, 2009).

Our goal in this perspectives piece is to stimulate conversations about the regulation and ethics of wildlife-focussed citizen science, using the UK context as a detailed case study. As Rasmussen and Cooper (2019) pointed out in their introduction to a special issue of *Citizen Science: Theory and Practice* devoted to ethics, there is no obvious drive from citizen scientists or regulators to talk about ethics in citizen science (at least not in the USA where these co-authors reside). Despite this, they noted that focussing on ethics not only shows a willingness to undertake a ‘healthy assessment of the field’, but is also sensible given that we should expect problems to arise at some point, as they would in any field. For example, while citizen science has not yet seen any major scandals involving researcher misconduct, such as the fabrication of data, there is always the potential for them to occur, especially when working practices have not been fully formalised. There have not to our knowledge been any scandals regarding animal welfare in wildlife-focussed citizen science, and citizen scientists may often possess a greater degree of skill and experience in trapping, handling, and marking animals than paid professionals with whom they collaborate (see Eitzel et al., 2017; Pocock et al., 2014). However, because wildlife-focussed citizen science involves direct engagement with animals, it potentially poses risks to animal welfare. Like other biodiversity conservation work, it may also involve important ethical considerations, including how to balance biodiversity conservation, the welfare of individual animals, and the values and rights of indigenous peoples such as customary hunting rights (Wehi & Lord, 2017) and non-Western understandings of animal welfare and ethics (Callicott, 1994; Doerfler & Peters, 2006; Linzey & Linzey, 2018). Such considerations arise, for example, in the case of subsistence hunting of narwhals (*Monodon monoceros*) and other species of conservation concern by indigenous groups in the Arctic, where indigenous hunters may be involved in recording the presence of wildlife or catching animals for tracking (Flora & Andersen, 2016). Following other similar work exploring ethics in citizen science (see other contributions to the 2019 *Citizen Science* special issue, and Resnik et al., 2015; Riesch & Potter, 2014), we therefore see a need to engage in discussions about ethics and regulation in wildlife-focussed citizen science.

We focus on *ethics* and *regulation* together because ethical standards inform the development of regulations, and regulations are in turn are deployed as tools for promoting ethical behaviour. For example, laws are deployed as instruments for protecting animal welfare, although in general domestic animals have received more attention in terms of welfare regulations than free-living wildlife (Dawkins & Bonney, 2008; Harrop, 1997, 2011, 2013). This pattern arguably follows a broader trend of wild animal welfare being relatively neglected as a subject of attention among wildlife researchers (e.g. see Cattet, 2013) and philosophers (e.g. see Paquet & Darimont, 2010). Wildlife law is also shaped by cultural practices, with Harrop (1999, 2013) arguing that the development of wild animal welfare and conservation law has been protracted by hunting interests, with hunting with hounds, for example, only banned in the UK in 2004. Wildlife law in countries with a history of colonisation may also fail to account for indigenous subsistence and customary hunting, such as in Aotearoa (the Māori name for New Zealand; Ruru et al., 2017; Wehi & Lord, 2017). Thus, laws are not only conceived of as tools for promoting ethical standards (such as humane treatment of animals), but are also shaped by philosophical, cultural, and historical approaches to ethics.

Regulations intended to promote or enforce ethical behaviour can be thought of as having varying levels of formality, ranging from ‘command and control’ regulations involving law and government enforcement, through to more informal and non-binding forms of regulation (Gorwa, 2019). Examples of the latter include: voluntary codes of conduct, such as those intended to promote ethical behaviour in companies (Gorwa, 2019; Hodges, 2015); internally developed and encouraged standards, within professions (e.g. the legal profession: Wendel, 2001) or institutions, such as via the promotion of ‘cultures of care’ within animal research facilities (Greenhough & Roe, 2018); pressure from communities affected by corporate behaviour, such as in response to pollution (Pargal et al., 1997); and the use of behaviour-change tools such as covert messaging to structure people’s choices towards a desired outcome, commonly known as ‘nudge’ (Baldwin, 2014; Jones et al., 2014; Whitehead et al., 2019).

Both formal and informal regulations have been proposed for encouraging ethical behaviour in citizen science. For example, Rasmussen (2019) suggested the use of best practice guidelines and training resources to help to prevent research misconduct, and Guerrini et al. (2018) proposed changes to laws and the creation of codes of conduct for protecting participants and intellectual property, and promoting scientific integrity in citizen science (see also Cooper et al., 2019). While some attention has been paid to the ethical and regulatory challenges faced by professional wildlife researchers (e.g. Cooke et al., 2016; Curzer et al., 2013; Russow & Theran, 2003; Sikes & Paul, 2013), few commentators have reflected on how formal and informal regulations work, and ought to work, for citizen scientists undertaking work with wildlife. This situation could, in part, reflect a reality that much citizen science receives little formal regulatory oversight (Rasmussen, 2019, p. 5). For example, Rasmussen (2019, p. 2) observed that citizen science lacks an overall approach to ensuring research integrity, in part because its ‘decentralized, open-access ethos’ means fewer formalised organisational processes such as institutional ethical review. In other words, citizen science may face less oversight than the work of professional scientists, in part because citizen science is inherently a decentralised activity involving members of the general public.

We reflect on the opportunities and challenges of regulating wildlife-focussed citizen science from a UK perspective. While some details are therefore necessarily country specific, we use them to reflect on questions and proposals that are applicable to citizen science internationally. The UK offers a particularly interesting case study because regulation of animal (including free-living wildlife) research in the UK is often described as particularly comprehensive.

We base our examination of the UK context on personal knowledge and experience. S.J.R. is a professional wildlife researcher and a member of a university Animal Welfare Ethical Review Board (AWERB); J.L. is the head of department of the National Wildlife Management Centre (NWMC) and has worked in the field of animal welfare and wildlife research ethics for over 20 years; and R.D. is an experienced citizen scientist at home and abroad, and chairs a national bird surveys committee. We also draw on qualitative research undertaken by A.P. and B.G. as part of *The Animal Research Nexus* (AnNex: https://animalresearchnexus.org/), a collaborative, interdisciplinary project investigating social and ethical questions related to animal research in the UK. We draw in particular on one sub-strand of the AnNex project which focusses on non-laboratory research in farms, fisheries, veterinary clinics, zoos, and wildlife field sites. While the focus of the research was primarily research regulated under the Animals (Scientific Procedures) Act (A[SP]A) (1986), it also encompassed non-A(SP)A wildlife research by citizen scientists. Semi-structured interviews were conducted with 30 people, and 24 lengthy informal conversations with others; five of these conversations focussed almost exclusively on wildlife-focussed citizen science and a further 16 on wildlife research. Participant-observation was also conducted during visits to research projects and relevant events, such as a conference focussed on wildlife citizen science. All interviews were conducted with the written consent of participants, and the research was approved by the University of Oxford’s Central University Research Ethics Committee (CUREC) (Reference Number: SOGE 18A-7). This qualitative research was not intended to capture views representative of wildlife researchers and citizen scientists, but rather to explore in-depth perspectives on key themes, including ethics and regulation. Analysis of interview transcripts, field notes and relevant documents was conducted using qualitative data analysis software NVivo. In addition to these research activities, the AnNex project involved running a workshop on non-laboratory research (see Animal Research Nexus, 2019) and a panel discussion on regulation of wildlife citizen science; S.J.R., J.L. and R.D. presented at both of these events.

This piece, and the panel discussion on citizen science regulation, were inspired by the ways in which regulation and ethics in wildlife-focussed citizen science emerged as key themes in the qualitative research and workshop on non-laboratory research. In particular, research and workshop participants expressed a range of views on whether there is a need for further regulation of hands-on work with free-ranging animals by citizen scientists (e.g. capturing, handling, and marking animals), given that some work (depending on the species) requires minimal licensing, training, and justification in the UK. For example, some small mammal trapping using Longworth traps, and trapping of some larger species such as red foxes (*Vulpes vulpes*), does not require licences. Some argued for the merits of extending the A(SP)A to cover a greater proportion of wildlife work by citizen scientists. Others reflected on the already complex legal landscape affecting wildlife research in the UK, and the risk that over-regulation would reduce the amount of citizen science taking place, suggesting that further formal regulation is undesirable. If no formal regulation was introduced, the question is then: How might we encourage, and potentially enforce, ethical behaviour in the absence of regulation? Our goal in this piece is not to argue from either of these positions, but rather to consider how and why these questions have arisen, some proposals for answering them, and the implications of these proposals. In addition, we propose three steps that could be taken to progress conversations about formal and informal regulation of wildlife-focussed citizen science in the UK and internationally.

## 2 Regulation of Wildlife-Focussed Citizen Science: The Key Sources of Debate in the UK

To begin, we briefly describe wildlife research legislation in the UK and the key debates surrounding it (see Harrop, 1997; Lane & McDonald, 2010 for further details on these laws). These laws, and relevant points about them in relation to capture and handling of wildlife by citizen scientists, are summarised in Table 1.

First, the A(SP)A (1986) is implemented and enforced by the HO. The A(SP)A regulates work with animals (vertebrates and cephalopods) undertaken for a ‘scientific or educational’ purpose, which meets or exceeds the ‘lower threshold’, defined (since 2013) as causing the animal ‘a level of pain, suffering, distress or lasting harm equivalent to, or higher than, that caused by inserting a hypodermic needle according to good veterinary practice’. While science is not explicitly defined within the A(SP)A or its associated guidance, specific activities are excluded from its remit. In the most recent revision of the A(SP)A these include ‘the ringing, tagging or marking of an animal’ or other procedures undertaken ‘for the primary purpose of enabling an animal to be identified, provided that it causes only momentary pain or distress (or none at all) and no lasting harm’, procedures commonly used by citizen scientists working with wildlife (for above see Home Office, 2014, pp. 10–12). In addition, the A(SP)A does not cover the capture of wild animals, although guidance indicates that animals must be captured by a ‘competent person using a method which does not cause the animal avoidable pain, suffering, distress or lasting harm’ (ASRU, 2016, p. 22). Similar purpose- and harm-based criteria restrict the scope of local laws related to animals in science across Europe, following the introduction of EU Directive 2010/63/EU on the protection of animals in science (Lindsjö et al., 2019; NORECOPA, 2017).

Based on our qualitative research and prior knowledge, we are aware that citizen scientists are commonly involved in assisting with the trapping and handling of animals in research regulated under the A(SP)A. While their involvement may be crucial for the success of A(SP)A-regulated projects, we understand that citizen scientists do not themselves commonly hold licences under the A(SP)A, although there are few statistics available to substantiate this statement. However, questions have been raised by some research and event participants about whether certain activities commonly undertaken by citizen scientists, which are not regulated under the A(SP)A, do in fact meet the criteria for inclusion under the Act (see also NORECOPA, 2017). Such questions may arise, in part, because determining the effects of certain trapping or marking methods on animals can be challenging. To give an example of the complexity involved in determining whether methods of marking exceed the ‘lower threshold’, the effects of biotelemetric tracking devices on bird flight are determined by a wide range of factors, such as species identity and tag placement as well as its mass and size (Vandenabeele et al., 2012, 2014). Considering obvious impacts on avian locomotion, such devices potentially affect other aspects of avian biology such as breeding performance and foraging success to name but two (see Geen et al., 2019; Vandenabeele et al., 2011 for reviews). For these reasons, the pragmatic ‘3% rule’ (i.e. tags should be at most 3% of the body mass of the bird on which they are deployed) is used widely in field ornithology but is no guarantee that deployment is free from tag-induced adverse effects (Geen et al., 2019). Recent research has suggested that tags weighing only 3% of avian body mass may result in a 4.67%–5.71% increase in energy expenditure during flights by seabirds (Vandenabeele et al., 2012). Thus, determining the circumstances under which tagging birds might exceed the threshold for inclusion under the A(SP)A is a complex task (see Wilson, 2017). Accordingly, the HO advises that although 3% of body mass is a commonly accepted maximum for tag mass for birds, it also specifies that ‘[w]eight alone should not be the only criterion used to assess the potential harms’ and that the overall welfare effects of tags should be taken into account in assessing whether use of a tag meets or exceeds the lower threshold (ASRU, 2016, p. 46). Furthermore, research and event participants have indicated that there is close collaboration between the HO and the BTO—which oversees bird ringing and tagging—about when tagging should fall under the A(SP)A. This, in turn, led some participants to argue that research on birds is exemplary in this respect and that citizen science on birds is more carefully regulated than that involving many other animal taxa.

This brings us to a second key source of regulation for wildlife research, the WCA (1981), which is the primary mechanism through which wildlife in Great Britain (it does not extend to Northern Ireland) is protected. Under the A(SP)A, only certain species are ‘protected’, namely vertebrates (except humans) beyond a certain stage of development, and any living cephalopod. Similarly, the WCA only classifies certain species as ‘protected’ for the purpose of the Act. Confusingly, however, ‘protection’ applies to different sets of species depending upon which legislative machinery (e.g. the A[SP]A, the WCA, other regulations such as the AWA [2006]) is being applied. Under the WCA, it is illegal to kill, injure, or possess intentionally a protected animal without a licence to do so. The WCA offers three types of licence: individual licences, which are granted on a case-by-case basis; general licences, which do not require individuals to apply for them but do specify eligibility criteria for their use (e.g. there is a general licence for catching shrews [Soricidae]: Natural England, 2018); and class licences, which fall in between the other two in that they require registration and evidence of competence but no full licence application. All ‘wild’ birds (but not gamebirds, which fall under their own Act) are protected generally under the WCA, although there are various general licences relating to birds. For example, certain general licences allow farmers, gamekeepers etc. to capture, including via the use of decoy birds in Larsen traps, and kill birds such as corvids (Corvidae) for a number of purposes (e.g. reducing loss of gamebird eggs and chicks, promoting public safety) if no other alternatives are available or effective (Natural England, 2020a, 2020b). Licences for this are issued by SNCOs: Natural England, Scottish Natural Heritage, and Natural Resources Wales. Licences specifically for catching birds for ringing purposes, and the use of tracking devices, are issued by the BTO with delegation from these SNCOs. The SNCOs are devolved and, we have been informed by research participants, operate slightly differently in different countries within the UK. Meanwhile, only certain mammals are protected under the WCA, with many common mammals (e.g. the European rabbit [*Oryctolagus cuniculus*], the red fox, the wood mouse [*Apodemus sylvaticus*]) having no specific protection under this or any other UK wildlife laws (Harrop, 1999).

Even species that are not protected under the WCA or other wildlife laws are covered by the AWA, which focusses on both preventing animal cruelty and ensuring that the welfare needs of an animal are met (see also Lane & McDonald, 2010). The AWA applies to any animal under human control, including those caught in a trap or temporarily restrained for marking. The AWA has recently been used successfully to prosecute someone for drowning a grey squirrel (Usherwood, 2010), indicating that it can be used in wildlife cases. Some common identification techniques (e.g. ear notching and microchipping) are technically ‘mutilations’ under the AWA, but they are explicitly permitted under the Mutilations (Permitted Procedures) (England) Regulations (2007) (Natural England, 2010).

The EU regulation on invasive alien species (1143/2014), introduced in 2014, can further complicate wildlife research by banning the release (including after capture) of the invasive alien species listed (e.g. Egyptian geese *Alopochen aegyptiaca*, grey squirrels), although permits are available for some activities for research purposes. Yet, many wildlife research projects are premised on a catch–release– observe/track model. This regulation can not only make research on certain animal species problematic, but can also mean that researchers and citizen scientists may be required to kill any non-target invasive species they inadvertently catch (i.e. ‘bycatch’).

The speciesism inherent in the WCA, and various other wildlife laws such as species-specific acts (e.g. The Deer Act [1991], The Protection of Badgers Act [1992]) and the EU regulation on invasive alien species, are clearly shaped by a variety of social and political factors. To name a few, Harrop (1999) identified hunting and economic interests as especially important in shaping which species are protected under wildlife laws, in the UK and elsewhere. In countries with a history of colonisation, subsistence and customary hunting by indigenous groups may be at odds with wildlife laws. For example, Wehi and Lord (2017) observed that customary hunting of *taonga* (i.e. highly valued) bird species by the Indigenous Māori is often not permitted by law in Aotearoa/New Zealand. Other forces potentially at play in shaping which wildlife species are protected by law include political campaigns led by non-governmental organisations (NGOs), and ‘accident, political expediency, [and] the media-driven power of aesthetics’ (Harrop, 1999, p. 694). One might also look to other cultural factors as influencing wildlife laws. Birds have featured prominently in arts, folklore, spirituality, healing practices, and other customs around the world (Tidemann & Gosler, 2010). For example, for Māori the now-extinct huia (*Heteralocha acutirostris*) was especially revered, with their feathers worn by chiefs of distinction during specific ceremonies and when entering battle (Houston, 2010). In the UK, birds are regarded as an especially ‘charismatic’ taxon (Lorimer, 2015). However, the cultural heritage and contemporary public perception of birds may vary by species; Eurasian magpies (*Pica pica*), for example, have historically been viewed as thieves and harbingers, and today tend to be less popular than other garden birds in the UK (see Hopper et al., 2019 for review). Overall, however, birds are a well-known and widely revered taxon in the UK and other ‘Western World’ countries such as the USA, with birdwatching and bird protection in both countries enjoying long histories (Dunlap, 2012; Lewis, 2012; Lorimer, 2015; Moss, 2013). Indeed, Dickinson et al. (2010) pointed out that alongside astronomy, ornithology is the Western scientific field with the largest body of ‘amateur experts’ and the longest history of volunteer engagement in research.

It is abundantly clear that the result of these laws is that citizen scientists in the UK working on wildlife are much more closely regulated in some species compared with others. For example, bird ringing licences from the BTO require extensive training, including in methods for trapping and the handling of birds, before ringers can act autonomously. However, some non-avian species receive little or no protection under the WCA and other wildlife laws. It is these situations that we find have attracted most concern amongst research and event participants. They worry that even if most citizen scientists undertaking trapping and marking are experienced and take care to attend to animal welfare, if there are no systems in place to check, less careful work could also theoretically occur. Similar concerns have been raised about patchy regulation of wildlife trapping and marking elsewhere in Europe, with some therefore recommending that decisions around all such work, irrespective of purpose, should be made with input from central animal research authorities (see Lindsjö et al., 2019; NORECOPA, 2017 for related discussions in Sweden).

Amongst our research and event participants, concern has been directed at forms of trapping perceived as particularly high risk. One mammalian example is the use of Longworth traps for capturing small mammals, with shrew mortality ranging from 10% to 93% (Shonfield et al., 2013). Indeed, trapping in general is widely perceived as the most stressful part of research for a wild animal, with the level of stress experienced by the animal likened to being pursued and captured by a predator (Wilson et al., 2019). Negative effects may also persist after capture. For example, myopathy, which is caused by a build-up of lactic acid in muscles and is particularly common in large mammals such as deer (Cervidae) following prolonged pursuit and handling, may take as much as a week to manifest and therefore cannot be detected during capture (Lane & McDonald, 2010). In a study of the effects of capture (using leghold snares, helicopter darting, or barrel traps) on grizzly bears (*Ursus arctos*) and American black bears (*Ursus americanus*), Cattet et al. (2008) demonstrated that bears’ mobility decreased after capture, only returning to normal levels after 3–6 weeks. Furthermore, they demonstrated that age-specific body condition tended to be lower among bears caught twice or more than among those only trapped once, with the magnitude of the effect directly proportional to the number of times caught; older bears showed the greatest adverse effects. Repeated capture can therefore have negative effects on animal welfare.

A further worry about unregulated wildlife trapping and marking is directed at work that is not obviously connected to any research projects or expansion of knowledge but rather perceived as undertaken primarily for recreational purposes. Arguably, such research would not meet any common definition of ‘citizen science’, since although citizen science can offer enjoyable opportunities to socialise and spend time in the outdoors (Dickinson et al., 2012), recreation is rarely viewed as an important central goal of projects labelled ‘citizen science’ (see Eitzel et al., 2017; Riesch & Potter, 2014 for more on ongoing debates about defining citizen science). That said, recreational anglers may be enlisted to collect data used for managing fisheries (Eden, 2012) or assessing the effects of repeated capture on fish welfare (Thorstad et al., 2020), so the distinction between recreation and citizen science can be hazy. In essence, the concern expressed by research participants was that some projects lacked sufficient benefits (e.g. contributions to the theoretical and applied scientific knowledge base) to outweigh the harms (e.g. to individual animal welfare). This worry links with a broader concern about citizen science—that if data quality is poor, projects may not be justified (Elliott & Rosenberg, 2019). While not necessarily required for securing licences under the WCA, rigorous harm-benefit analysis is required for securing licences under the A(SP)A (ASC, 2017; Davies, 2018). While many wildlife researchers we spoke with complained about the investment required for securing licences under the A(SP)A, they also commonly praised the A(SP)A for forcing them to think carefully about how and why they want to carry out research due to its emphasis on harm-benefit analysis and the 3Rs, which encapsulate a commitment to *Reduce* the number of animals used, *Refine* methods so as to minimise harm, and *Replace* animal research with alternatives whenever possible. For example, one interviewee referred to harm-benefit analysis, and the requirement under the A(SP)A to specify actions to be taken if something goes wrong (e.g. the point at which an animal should be euthanised), as ‘the good bits of A(SP)A’ (interview with veterinary researcher, 21 November 2018). That said, some of our research participants have also suggested that training requirements for undertaking HO-regulated work leave something to be desired, with HO-accredited training potentially completed in weeks, compared with the months or years of supervision required before BTO ringers can work independently.

As a result of these considerations, some participants in our research and events have made the case that the A(SP)A ought to be extended to cover trapping and all marking of wildlife for scientific research purposes, and potentially also for ‘identification purposes’ if identification is regarded as a method (undertaken for either science or husbandry) rather than a purpose in its own right (interview with wildlife researcher, 12 March 2019). This move would mean that all citizen science involving trapping and marking of wildlife— at least, that which could be viewed as undertaken for a ‘scientific purpose’—would require the same attention to harm-benefit analysis, animal welfare, the 3Rs, and training as required for any other research licensed under the A(SP)A. However, published literature and research participants have highlighted several issues with this proposal.

**Figure 1:**
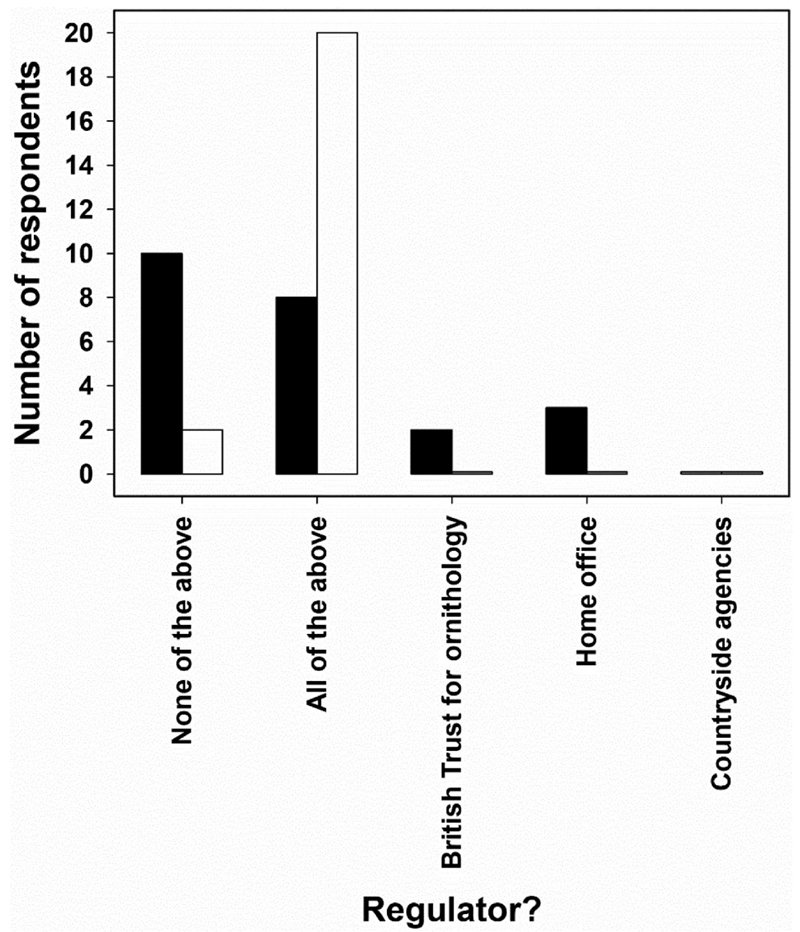
Audience responses before (filled; *N* = 23) and after (open; *N* = 22) a panel discussion on the regulation of wildlife research by citizen scientists. The public event was held at the Oxford Museum of Natural History on 9 November 2019. Responses were to the question, ‘Who regulates wildlife research by citizen scientists?’

One concern is that introducing still more formal regulation to what is already a permit-heavy area may not be ideal. Paul and Sikes (2013) described a feeling of wildlife researchers ‘running the permit maze’ in the USA, and similar ideas were expressed by research and event participants working in the UK. Furthermore, before and after presentations by S.J.R., R.D., and J.L. at our panel discussion on regulation of wildlife citizen science, we elected to ‘poll’ the audience using Mentimeter (software which enables live surveys of audiences) on several questions, including: Who regulates wildlife research by citizen scientists? Responses indicated a dramatic shift in audience understanding when asked before and after the three presentations and associated discussions (Figure 1), suggesting that knowledge of wildlife research regulation is patchy, even in an audience in which nearly half indicated that they were involved in wildlife research or regulation, and nearly a quarter were conducting citizen science.

Another interesting result of our polling derived from our final question to the audience at the end of the event: Should the amount of regulation of wildlife research change? The majority (11 people) indicated that it should stay about the same, while equal numbers (three people) voted for more or less regulation and one attendee remained unsure. We interpret this result as reflecting a more general theme emerging from our research: that few would like to see further regulation brought into this already crowded area of policy. Yet, gaps remain in wildlife regulations—such as the lack of licensing, training, and justification needed for some wildlife trapping and marking—while other areas are arguably over-regulated. For example, should a research activity that is regulated under the A(SP)A (e.g. blood sampling) be conducted on an invasive bird species such as the Egyptian goose, a researcher would require licences from the HO, an SNCO, the BTO, and the APHA, which will issue permits under the EU regulation on invasive alien species. To improve this situation, the Law Commission recommended in 2015 considerable simplification of wildlife law in the UK, which was described as a ‘complex patchwork of overlapping and sometimes conflicting provisions’ (Law Commission, 2015, p. 3, paragraph 1.8).

Several participants have expressed concern that extending the A(SP)A to cover trapping might discourage citizen scientists from engaging in wildlife research at all, thereby eliminating the benefits of such work. Perhaps more plausibly, such a move could drive citizen science involving trapping and marking of wildlife underground, which could prevent fruitful collaborations between citizen scientists and professional researchers, whose institutions would be required to follow the law. Our research has indicated that because HO licences require substantial financial costs (e.g. for training, application fees, and potentially upgrading equipment or facilities) and entail much time to secure, some organisations and individuals actively steer clear of engaging in any research activities that might require such licences. For example, participants based in zoos and teaching-focussed agricultural and land-based colleges have indicated that they feel their institutions lack the appropriate expertise and other resources (e.g. time, money) to secure the appropriate licences for such work. For these reasons, an interviewee involved in a citizen science organisation proposed that extending the A(SP)A in this way would ‘scupper’ much citizen science research, since licensing is ‘quite difficult’ for those not associated with an institution equipped to handle it (interview, 3 January 2020).

If the Law Commission’s recommendation of overhauling and simplifying the UK’s wildlife law was followed, extending the A(SP) A in this way may not be necessary, although some may also wish to see a greater emphasis on the 3Rs and harm-benefit analysis introduced into wildlife laws as part of this overhaul. If this does not happen, extending the A(SP)A could represent a double-edged sword, since it could simultaneously enhance ethical oversight of citizen science and prevent much citizen science from taking place. In short, extending the A(SP)A to cover wildlife trapping and marking would likely raise, rather than lower, the bar to entry for citizen scientists and thereby undermine citizen science’s potential benefits. In addition to enabling professional researchers to undertake research that could not occur without the help of citizen scientists, perceived benefits of citizen science include fostering a sense of connection and engagement with science and with the natural world, and, at least if one adopts Irwin’s (1995) concept of ‘democratic citizen science’, giving non-professional scientists a stake and a say in how science is conducted (see Cooper & Lewenstein, 2016; Resnik et al., 2015 for overviews).

The results of research involving citizen scientists can also have important scientific and conservation implications. For example, since 1990, the ornithological societies of the three UK military services—namely the Army Ornithological Society (AOS, of which co-author R.D. is Chairman), with assistance from the Royal Air Force Ornithological Society (RAFOS) and the Royal Naval Birdwatching Society (RNBWS)—have conducted a longterm population monitoring study of the seabird community on Ascension Island in the South Atlantic, one of 14 UK Overseas Territories. In 2008 they teamed up with S.J.R. (a co-author of this paper) at the University of Birmingham and since then they have published 16 peer-reviewed scientific papers and a book (e.g. Bolton et al., 2012; Reynolds et al., 2019). Working closely also with the Ascension Island Government and the University of Exeter (Cornwall Campus) in the UK, they have also provided invaluable seabird tracking data towards the August 2019 designation of the largest marine protected area (MPA) in the entire Atlantic and taken ownership of the Biodiversity Action Plan (BAP) on the island, providing a Species Action Plan (SAP) for the sooty tern (*Onychoprion fuscatus*) and the brown noddy (*Anous stolidus*).

Had the A(SP)A been extended, this specific project may not have been affected, since the A(SP)A does not legally apply in UK overseas territories; furthermore, the involvement of a university-based research group would have enabled citizen scientists to secure licences under the A(SP)A if necessary. However, other work undertaken by the AOS and other citizen science organisations in the UK would likely not have proceeded. Extending the A(SP)A could therefore help to ensure ethical oversight and harm-benefit analysis of citizen science, but could also in practice prevent much citizen science from taking place, or drive it underground. The question, then, becomes: Is there is an alternative, preferable way forward?

## 3 Moving Forwards

To conclude, we highlight three steps that we propose should be taken to progress discussions of ethics and regulation in wildlife-focussed citizen science, as illustrated in Table 2.

We propose that these three steps may also be applicable to other countries, although the nature of each step may differ. For example, countries with indigenous peoples will need to consider indigenous rights and management responsibilities in considering any formal regulatory reforms affecting citizen science. Still, broadly these steps could be applied internationally with effectiveness. We do not offer a strict timeline for these steps but we suggest that where possible they are best undertaken in the order specified in Table 2. They should begin as soon as possible; in the UK our research has highlighted a desire among many research and event participants to resolve outstanding issues as a matter of priority. However, each step involves a slightly different set of actors: step 1—SNCOs and citizen science groups; step 2—government regulators; and step 3—citizen science societies and groups, institutions engaged in citizen science (e.g. NGOs) or which administer citizen science projects (e.g. Zooniverse: www.zooniverse.org), professional researchers, and citizen scientists themselves where participatory approaches are adopted. Because a variety of actors are involved, citizen science communities cannot accomplish all three steps on their own, and may be restricted to implementing only step 3 without support from government regulators.

We now describe in greater depth the justification for each step, and what resolutions could involve. The first step we propose is to take stock of the range and number of procedures currently undertaken by citizen scientists with wildlife (Table 2). Obtaining a sense of the scale of such work, and the level of harm this work causes to individual animals, could inform decisions about the most appropriate tools for regulation. For example, it has been proposed that extending the scope of the A(SP)A to include wildlife trapping by citizen scientists would result in a perhaps unworkably large number of additional people for the HO to regulate, most of whom would likely be doing work classified as ‘low risk’ in terms of animal welfare harms and public concern. To place this proposal in context, the BTO reported that 982,858 birds were ringed by licensed ringers in 2018 (Robinson et al., 2019). However, SNCOs collect but do not publish similar statistics on the number of, and the nature of research conducted on, non-avian animals under licence. Taking stock of citizen science activity in the UK would therefore require the publication of more comprehensive statistics by SNCOs. For species with minimal protection under UK wildlife law, gathering such information might also require liaising directly with local wildlife groups or via centralised citizen science organisations (Table 2). In other countries, similar steps may be required if the numbers of animals captured for citizen science are not already recorded or publicly reported.

Even if such statistics were available, we would still need to determine whether introducing formal regulation to citizen scientists would be desirable, hence our proposed second step of assessing the state of formal regulations and considering reforms (Table 2). This step comes with several issues and challenges. One is that citizen scientists would be unlikely to be directly involved in formal regulatory reform; decisions about citizen science would therefore be made in a top–down manner. This issue resonates with broader discussions around power dynamics in citizen science, such as the risk that citizen scientists will receive insufficient credit for their work (see Resnik et al., 2015; Riesch & Potter, 2014). Furthermore, resolving this step requires consideration of who would be tasked with regulation, and how this would affect their needs in terms of staffing and expertise, and their ability to regulate effectively. In addition, revising formal regulations would involve deciding on how to balance the perceived benefits of wildlife research carried out by citizen scientists—for example, encouraging public engagement with science and nature, and contributing to science and conservation—with the risks, particularly compromises to animal welfare. To an extent, this consideration reflects a more general problem of balancing species and ecosystem conservation with individual animal welfare (e.g. Beausoleil, 2014; Hayward et al., 2019; Paquet & Darimont, 2010; Ramp & Bekoff, 2015), and in particular how best to balance these concerns in formal regulation (Harrop, 2010).

One possible outcome of this second step is the conclusion that informal regulation is more desirable or effective than formal regulation for encouraging best practice and ethical behaviour in citizen science. The strengths of formal versus informal regulations have received considerable attention in relation to other areas of regulation and behaviour. To take one example, Hodges (2015) summarised key debates about regulation’s role in shaping ethical corporate behaviour. One significant problem is that formal regulation of various kinds does not always have the desired result of behavioural change. For example, actors might engage in ‘creative compliance’ whereby they follow the letter, but not the spirit, of the law (p. 2). Other scholars have highlighted a risk that regulations enforced through auditing become merely a ‘tick box’ exercise, with the audit process becoming decoupled from the qualities it is meant to assure and becoming an end in itself (e.g. Escobar & Demeritt, 2017; Power, 1999; Strathern, 2000). Meanwhile, Hodges (2015) argued that formal regulations may be ineffective because they are based on flawed understandings of human behaviour, such as the principle of deterrence (i.e. that punishment for undesirable behaviour will prevent similar behaviour in future) and the idea of humans as ‘rational actors’ who function in isolation. Formal regulation’s effectiveness may also be limited given that it tends to focus on deterring negative, rather than encouraging positive, behaviour. For these reasons, Hodges (2015) advocated for a holistic approach to encourage ethical behaviour in which formal regulatory systems are combined with a broader range of approaches, with the goal of encouraging corporations to ‘achieve and exceed compliance’ (p. 703) rather than simply deterring negative behaviour.

Informal regulatory mechanisms may therefore also play an important role in encouraging ethical behaviour, hence our proposed third step that informal regulations be considered as alternatives or supplements to formal regulations (Table 2). For instance, in the realm of animal research a ‘culture of care’ is increasingly encouraged. While the culture of care concept is mentioned in EU regulations and the A(SP)A guidance documents (ASRU, 2017; Greenhough & Roe, 2018), it is not strictly tied to formal regulation, and is perhaps best described as a kind of ‘workplace atmosphere’ in which care is encouraged for both animals and staff, and legal regulations are not just met but exceeded (Greenhough & Roe, 2018, p. 12). In other words, a culture of care is primarily a product of informal regulation through professional and institutional standards rather than formal regulation through laws and enforcement. Rasmussen (2019) made similar proposals for preventing misconduct amongst citizen scientists. Among other ideas, Rasmussen (2019, p. 5) suggested enlisting citizen science organisations to disseminate key resources, run tutorials, and encourage their members to follow best practice guidelines, with the goal of promoting a ‘culture of research integrity’ within citizen science. However, one potential weakness of this type of informal regulation is that institutional and professional cultures may develop harmful as well as positive norms (Wendel, 2001).

In addition to institutional or professional values and standards, covert manipulation of behaviour is also sometimes employed as an informal regulatory mechanism for encouraging desirable behaviour. ‘Nudge’ describes an approach of structuring people’s choices to lead them towards a desired outcome, such as supplying information and reminders, placing limitations on choices, and using covert framing strategies to encourage people to make healthy lifestyle choices (see Baldwin, 2014). One could imagine nudges in wildlife research taking the form of good practice guidelines included with every animal live trap sold, or voluntary codes of good practice in wildlife research. However, an important question to consider is: What exactly should citizen scientists working with wildlife be nudged towards? The philosophical underpinning of nudge is that of ‘libertarian paternalism’, in which choices are technically left open but citizens are encouraged to make the ‘right’ choice. However, as Baldwin (2014) pointed out, there is room for disagreement about right and wrong choices, even in cases where the nudge is simply intended to improve an individual’s wellbeing—for example, many people might choose to enjoy an unhealthy activity like drinking alcohol even if it shortens their life span. This covert manipulation of behaviour towards a specific desired outcome, disguised with a veneer of ‘freedom’, is one reason that ‘nudge’ remains controversial (e.g. see Leggett, 2014; Whitehead et al., 2019).

Similarly, there is room for disagreement about what constitutes ‘justified’ wildlife-focussed citizen science. For example, when it comes to assessing whether the harms to welfare of individual animals outweigh the benefits to conservation (e.g. whether catching animals and attaching tracking devices yields sufficiently useful data to be justified), we might expect people’s answers to vary. Similarly, there might be discrepancies in people’s culturally bound understandings of animal suffering and welfare, and their views on how to balance conservation with other values such as indigenous hunting rights. Nudging citizen scientists towards a specific answer would therefore involve arbitrating between different ethical stances. It would also involve determining the value of data collection intended to monitor wildlife, rather than to test a specific hypothesis. As Elliott and Rosenberg (2019) observed, such monitoring-focussed work has been disparagingly labelled ‘fishing expeditions’ or ‘stamp collecting’ by critics, although others (including the authors) argue that such work often makes important contributions to science (see also Eitzel et al., 2017). It may therefore be undesirable to nudge citizen scientists towards a specific answer if we hope to respect variation in ethical stances and perceptions of scientific value, and avoid a situation in which one party (e.g. regulators, a group of professional scientists, or non-indigenous decision-makers) determines what counts as ethically acceptable citizen science.

This is perhaps where more participatory forms of informal regulation should come to the fore. For instance, informal regulation can take the form of communities boycotting or protesting the work of companies, which may in turn shape corporate behaviour (Pargal et al., 1997). Taking a more participatory approach would also resonate with the more ‘democratic’, PAR-oriented, and ‘extreme’ strands of citizen science, which encourage public participation in shaping the goals and processes of science (Cooper & Lewenstein, 2016; Eitzel et al., 2017; Haklay, 2013; Riesch & Potter, 2014). More participatory approaches to promoting ethical standards could involve engaging with citizen scientists in deciding on harms and benefits of research from the outset. It could also involve encouraging citizen scientists towards a process rather than a result, such as directing them towards harm-benefit analysis rather than a specific position on the circumstances under which animal welfare outweighs the accumulation and application of scientific knowledge. This approach might be understood as resembling the A(SP)A which, while offering guidance on the kinds of factors to be considered in harm-benefit analysis, does not contain a predetermined position on the circumstances when scientific benefits outweigh harms to animal welfare, or vice versa. Thus, formal regulation can also potentially be process oriented and flexible. To give an example of participatory, process-oriented informal regulation, during a workshop on non-laboratory research we discussed the idea of creating a simple tool such as a mnemonic that could be disseminated to remind citizen scientists to think carefully about regulation, animal welfare, and harms and benefits associated with their research activities (Animal Research Nexus, 2019). This kind of informal regulation could help to promote consideration of ethics, harms, and benefits within citizen science communities while ensuring that citizen science could still readily proceed. It could also maintain the ‘democratic’ ethos of some citizen science via the process of co-producing ethical standards.

To conclude, we have argued in this piece that there is a need to talk about ethics and regulation in wildlife-focussed citizen science. In a context where trapping and marking certain species (e.g. various non-avian taxa) require minimal licensing, training, and justification in the UK and elsewhere (e.g. see Lindsjö et al., 2019; NORECOPA, 2017), there is a worry that even if most citizen scientists are experienced and attend to animal welfare, less careful work could theoretically occur if there are no systems in place to check. However, while some of our research and event participants have argued for increased formal regulation to minimise risks to animal welfare, others have counter-argued that this could make citizen science less accessible and eliminate its benefits. Informal regulation might therefore be considered. In order to respect variation in perceptions of what constitutes ‘justified’ research, it may be preferable to encourage citizen scientists towards a process of ethical evaluation rather than a result. Some wildlife-focussed citizen science (e.g. ringing birds under the BTO’s authority) is already thoroughly and formally regulated, but some others with little formal regulation might already encourage ethical behaviour in various ways, such as offering best-practice guidelines (e.g. a booklet on live trapping small mammals published by the Mammal Society: Gurnell & Flowerdew, 2006). Despite this, we think it is nonetheless advisable to encourage and promote conversations about ethics and regulation in citizen science, as a way of undertaking a ‘healthy assessment of the field’ (Rasmussen & Cooper, 2019, p. 1). We have proposed three steps that could be taken to progress these conversations, both in the UK and internationally (Table 2).

## Supplementary Material

Glossary

## Figures and Tables

**Table 1:** Summary of key laws regulating animal research and citizen science in the UK, and relevant points about them relating to capture and handling of wildlife by citizen scientists

Law	Regulator	Summary
Animals (Scientific Procedures) Act (A(SP)A)	Home Office (HO)	Regulates invasive animal research undertaken for scientific purposes. Does not cover capture of wild animals. Does not cover ringing and marking if the primary purpose is ‘identification’ and it causes only momentary pain or distress
Wildlife and Countryside Act (WCA)	Statutory Nature Conservation Organisations (SNCOs) British Trust for Ornithology (BTO) regulates bird ringing	Regulates disturbance, killing, and possession of wildlife. Only certain species are protected under the WCA or other species-specific laws. Capture and handling may require extensive training (e.g. bird ringing under the BTO) but also may require no training or licence
Animal Welfare Act (AWA)	Enforced by various organisations, e.g. the Department for Environment, Food and Rural Affairs (DEFRA), the Royal Society for the Prevention of Cruelty to Animals (RSPCA)	Prohibits animal cruelty and ensures animal welfare needs are met, for any animal under human control. Can apply to wildlife during capture and handling
EU regulation on Invasive Alien Species (1143/2014)	The Animal and Plant Health Agency (APHA) issues permits in the UK on behalf of DEFRA	Outlines prevention, detection, eradication and management of invasive species across the EU. Requires that certain invasive species be killed if caught (e.g. grey squirrels *Sciurus carolinensis*)

**Table 2:** Three proposed steps to progress discussions of ethics and regulation in wildlife-focussed citizen science

Step	Justification	Possible resolutions
1. **Take stock** of wildlife-focussed citizen science in terms of numbers and harms to animal welfare	Information required before assessing practicality and desirability of formal and informal regulations	Collaborate with SNCOs (or other within-country equivalent) and citizen science organisations to compile publicly available statistics
2. Assess the state of **formal regulations** and consider reforms	Formal regulations can play a role in encouraging ethical behaviour	Simplify laws according to Law Commission’s advice, or extend the A(SP)A (or other within-country equivalent) to cover wildlife trapping and marking more comprehensively
3. Consider **informal regulations** as alternatives or additions to formal regulations	Formal regulations may be undesirable or impractical, or best supplemented with informal regulations	Encourage institutional or sector-wide standards (e.g. via dissemination of best practice guidelines and training resources), and take a participatory or process-oriented approach (e.g. via creation of a mnemonic) to encourage reflection on animal welfare and promote harm-benefit assessment

## Data Availability

By agreement with the Wellcome Trust and research participants, anonymised interview transcripts will be deposited in the UK Data Archive based at the University of Essex (https://www.data-archive.ac.uk) after a period of 10 years from the completion of the Animal Research Nexus Project in 2022, except in cases where participants have explicitly opted out of this arrangement.
