# Supplementary material for: Getting to grips with wildlife research by citizen scientists: What role for regulation?
Source: People Nat (Hoboken). Author manuscript; Available in PMC 2021 Feb 3. (PMC7116685; doi:10.1002/pan3.10151)
Supplement: Glossary [file EMS107979-supplement-Glossary.docx]

# GLOSSARY

**3Rs** Replacement, Reduction, and Refinement. Guiding principles to promote ethical use of animals in research after book by Russell and Burch (1959) “The Principles of Humane Experimental Technique”.

**A(SP)A** Animals (Scientific Procedures) Act (1986). Key regulation in the UK governing the use of animals in scientific research, largely through licensing.

**AOS** Army Ornithological Society.

**AWA** UK Animal Welfare Act (2006). Prohibits animal cruelty and ensures animal welfare needs are met, for any animal ‘under the control of man’.

**AWERB** Animal Welfare Ethical Review Board. Usually based in a UK research institution like a university, company, or government body. The AWERB reviews A(SP)A license applications before they are sent to the **Home Office** for approval, revision or rejection.

**BAP** Biodiversity Action Plan.

**BTO** British Trust for Ornithology. Regulates bird ringing under the **Wildlife and Countryside Act** on behalf of the Joint Nature Conservancy Council and country authorities (Natural England etc).

**DEFRA** UK Government Department for Environment, Food and Rural Affairs.

**HO** Home Office. UK Government Department responsible for regulating animal research.

**MPA** Marine Protected Area.

**NGO** Non-governmental organisation.

**PAR** Participatory Action Research. Approach used predominantly in the social sciences that aims to incorporate the expertise and views of participants in research design and practice.

**RAFOS** Royal Air Force Ornithological Society.

**RNBWS** Royal Naval Bird Watching Society.

**RSPCA** Royal Society for the Prevention of Cruelty to Animals.

**SAP** Species Action Plan.

**SNCO** Statutory Nature Conservation Organisation. Organisations in countries within the UK (Natural England, Scottish Natural Heritage, Natural Resources Wales) that administer licences under the **Wildlife and Countryside Act**.

**WCA** Wildlife and Countryside Act. Regulates disturbance, killing, and possession of wildlife.
